# Impact of Medication Regimen Simplification on Medication Administration Times and Health Outcomes in Residential Aged Care: 12 Month Follow Up of the SIMPLER Randomized Controlled Trial

**DOI:** 10.3390/jcm9041053

**Published:** 2020-04-08

**Authors:** Janet K. Sluggett, Ria E. Hopkins, Esa YH Chen, Jenni Ilomäki, Megan Corlis, Jan Van Emden, Michelle Hogan, Tessa Caporale, Choon Ean Ooi, Sarah N. Hilmer, J. Simon Bell

**Affiliations:** 1 Centre for Medicine Use and Safety, Faculty of Pharmacy and Pharmaceutical Sciences, Monash University, Melbourne, VIC 3052, Australia; janet.sluggett@unisa.edu.au (J.K.S.); ria.hopkins@monash.edu (R.E.H.); esa.chen@monash.edu (E.Y.C.); jenni.ilomaki@monash.edu (J.I.); choonean.ooi@monash.edu (C.E.O.); 2 School of Health Sciences, Division of Health Sciences, University of South Australia, Adelaide, SA 5005, Australia; 3 NHMRC Cognitive Decline Partnership Centre, Hornsby Ku-ring-gai Hospital, Sydney, NSW 2077, Australia; mcorlis@helpinghand.org.au (M.C.); JVanEmden@helpinghand.org.au (J.V.E.); mehogan@helpinghand.org.au (M.H.); sarah.hilmer@sydney.edu.au (S.N.H.); 4 Department of Epidemiology and Preventive Medicine, Monash University, Melbourne, VIC 3004, Australia; 5 Helping Hand Aged Care, Adelaide, SA 5006, Australia; tcaporale@helpinghand.org.au; 6 Kolling Institute of Medical Research, Royal North Shore Hospital, Northern Clinical School, School of Medicine, University of Sydney, Sydney, NSW 2050, Australia

**Keywords:** cluster-randomized controlled trial, medication regimen simplification, residential aged care, nursing homes, long-term care, medication administration, incidents, falls, mortality, hospitalization

## Abstract

In the SImplification of Medications Prescribed to Long-tErm care Residents (SIMPLER) cluster-randomized controlled trial, we evaluated the impact of structured medication regimen simplification on medication administration times, falls, hospitalization, and mortality at 8 residential aged care facilities (RACFs) at 12 month follow up. In total, 242 residents taking ≥1 medication regularly were included. Opportunities for simplification among participants at 4 RACFs were identified using the validated Medication Regimen Simplification Guide for Residential Aged CarE (MRS GRACE). Simplification was possible for 62 of 99 residents in the intervention arm. Significant reductions in the mean number of daily medication administration times were observed at 8 months (−0.38, 95% confidence intervals (CI) −0.69 to −0.07) and 12 months (−0.47, 95%CI −0.84 to −0.09) in the intervention compared to the comparison arm. A higher incidence of falls was observed in the intervention arm (incidence rate ratio (IRR) 2.20, 95%CI 1.33 to 3.63) over 12-months, which was primarily driven by a high falls rate in one intervention RACF and a simultaneous decrease in comparison RACFs. No significant differences in hospitalizations (IRR 1.78, 95%CI 0.57–5.53) or mortality (relative risk 0.81, 95%CI 0.48–1.38) over 12 months were observed. Medication simplification achieves sustained reductions in medication administration times and should be implemented using a structured resident-centered approach that incorporates clinical judgement.

## 1. Introduction

Increasing multimorbidity is contributing to increasing medication regimen complexity in residential aged care facilities (RACFs), with more than one-third of residents having five or more daily medication administration times [1,2,3,4]. Multiple administration times, modified dose forms (e.g., crushed tablets) and special administration instructions all contribute to medication complexity, which may place residents at risk of clinical incidents and poorer health outcomes [5,6,7,8,9]. 

Associations between regimen complexity and mortality, hospitalization and falls are not well explored. One population-based study observed associations between regimen complexity and mortality over 3 years for both community and RACF-dwelling older people [9]. A prospective study of Australian RACF residents identified an association between increased medication regimen complexity and 12 month hospitalization [6]. Evidence in community-dwelling older people varies considerably [10], with studies showing either no association [11,12,13,14] or significant associations [7,8,15,16] between discharge medication regimen complexity and rehospitalization. Associations between regimen complexity and falls have not been reported in any setting.

Medication regimen simplification through consolidating dose times or selecting combination medications, where available, may reduce complexity without changing overall therapeutic goals [17,18,19,20,21]. The number of administration times has been identified by community-dwelling individuals as more burdensome than the overall number of medications or medication classes [22]. In RACFs, decreasing administration times may result in fewer medication rounds and reduced burden of medication taking for residents, while allowing redirection of nursing time to other direct care activities [23,24]. 

In the SImplification of Medications Prescribed to Long-tErm care Residents (SIMPLER) cluster-randomized controlled trial (RCT), structured medication regimen simplification significantly reduced the number of daily medication administration times in the intervention arm at 4 months post-study entry [25]. Residents in the intervention arm received a structured medication simplification intervention using the Medication Regimen Simplification Guide for Residential Aged CarE (MRS GRACE) [24,26]. Consisting of key considerations for clinicians to apply when assessing potential to simplify medication regimens, this implicit (judgement-based) tool was developed by a multidisciplinary panel and validated for use in RACFs [26]. The trial identified a mean of four administration times per day at baseline (standard deviation (SD) 1.8) among 242 participants, with 92 simplification opportunities identified for 62 of the 96 residents who received the intervention. We have previously shown a reduction in the number of administration times in the intervention arm at 4 months compared to the comparison arm (mean difference −0.36, 95%CI −0.63 to −0.09) [25]. No differences were observed between study arms in the rate of falls, hospitalizations or mortality at 4 month follow up.

In these pre-planned secondary analyses, we evaluated whether the impact of medication regimen simplification on the number of medication administration times observed at 4 months was sustained at 8 and 12 month follow up, and investigated the impact on falls, hospitalization, and mortality over 12 months.

## 2. Experimental Section

### 2.1. Study Design

The SIMPLER study was a non-blinded, cluster RCT involving residents from eight RACFs, also known as long-term care facilities or nursing homes, that were operated by a South Australian not-for-profit aged care provider. The RACFs were paired based on bed numbers and geography (regional or metropolitan), with one RACF from each pair randomized to the intervention arm by a pharmacoepidemiologist using SAS random number generator (SAS Institute, Cary, NC, USA). The previously published study protocol includes these secondary analyses [24]. English-speaking permanent residents prescribed at least one regular daily medication were eligible to participate, unless deemed medically unwell or estimated by RACF nurses to have less than three months to live. Recruitment occurred from 24 April to 23 October 2017, with written consent sought from residents, or from the resident’s guardian, next of kin or significant other if the resident was unable to provide informed consent. Research nurses, residents, RACF staff and other stakeholders were blinded to group allocation at the time of recruitment and baseline data collection but were unblinded beyond this point.

### 2.2. Intervention

The intervention was delivered at the individual resident level by an experienced clinical pharmacist who applied MRS GRACE to identify opportunities to simplify participants’ medication regimens [26]. This involved identifying opportunities to administer medications at the same time, use long-acting formulations in preference to short-acting formulations, and switch from multiple single-ingredient to combination formulations where possible, without changing the therapeutic intent of the regimen [26]. Where an equivalent combination product was not available in the same doses as the single-ingredient preparations, simplification was not suggested. The pharmacist outlined opportunities to simplify medication regimens in a report for RACF staff and the resident’s general medical practitioner (GP).

### 2.3. Review and Implementation of Simplification Recommendations by the GP and RACF Staff

The protocol by which the GP and RACF staff reviewed and implemented recommendations was endorsed by the aged care provider’s multidisciplinary medication advisory committee prior to study commencement. Senior registered nurses at the RACF were able to implement recommendations concerning changing administration times of existing medications. All recommendations involving medication changes, such as replacing single-ingredient medications with a combination product or switching from an immediate release to a long-acting formulation, required GP implementation. Residents allocated to the comparison arm received usual care. Residents in the intervention and comparison arms were eligible to continue to receive comprehensive residential medication management reviews (RMMRs) conducted by pharmacists in conjunction with GPs [27]. 

### 2.4. Outcomes

Outcomes were measured at the individual participant level. The present analysis focused on the secondary outcomes of the number of charted medication administration times over a 24 h period for regular medications at 8 and 12 months, and falls, hospitalizations and mortality over 12 months, post-study entry. Medication administration times were ascertained from data extracted from resident medication administration charts. Nutritional drinks, pro re nata (PRN, as required) or nurse-initiated medications, short-term therapies such as antimicrobials, or medications not administered daily were not considered when calculating the number of daily administration times. The same definition of regular medications was applied at 4 month follow up (primary SIMPLER study outcome) [25]. 

Hospitalizations and date of death, if applicable, were extracted from the RACFs electronic medical records system, and hospital length of stay was calculated. Falls were extracted from the aged care provider’s risk management and reporting software, which was uniform across all study sites. A fall was any event that resulted in a resident coming to rest inadvertently on the floor, ground or other lower level [28]. Falls were reported prospectively by RACF staff according to the organization’s Client Incident Reporting Policy. 

### 2.5. Covariates

Baseline demographic data collected for SIMPLER participants included age, gender and RACF length of stay. Medications associated with an increased risk of falls were defined in accordance with the Swedish National Board of Health and Welfare criteria and included psychotropics and medications that can cause orthostatic hypotension [29]. The following functioning and comorbidity assessments were collected from records or directly from residents by study personnel or RACF staff: the Charlson Comorbidity Index [30], the Dementia Severity Rating Scale [31], Part 1 of the Fall Risk Assessment Tool [32], the Katz Activities of Daily Living scale [33], the Mini Nutritional Assessment-Short Form [34], and the FRAIL-NH scale [35]. Part 1 of the validated Fall Risk Assessment Tool was completed by RACF nurses and considered recent falls (scored out of 8), medications (scored out of 4), psychological conditions (scored out of 4) and cognitive status (scored out of 4) to provide an overall fall risk score out of 20 [32]. Residents with a fall risk score of 5–11 were classified as being at a low risk of falls, residents with scores of 12–15 were classified as at a medium risk of falls, and residents with scores of 16–20 were classified as at a high risk of falls [32]. For fall and hospitalization outcomes, incident rates in the 12 months pre-study entry were considered as covariates.

### 2.6. Analysis

Constrained longitudinal data analysis (cLDA) models tested associations between the intervention and medication administration times. The estimated mean difference in administration times and 95% confidence intervals (CIs) were determined. Falls and hospitalizations are presented as incident rates, calculated per 1000 resident days contributed to the study, considering loss-to-follow up and hospital inpatient days, to mitigate survivorship bias. Linear mixed models assessed associations between the intervention and incident rate ratios (IRRs) for falls and hospitalizations, and mean difference in hospital length of stay, and relative risk was presented for mortality. 

Analyses were undertaken using an intention-to-treat approach. All models included RACFs as random effects to account for clustering. Overdispersion was tested, and negative binomial distributions were used to estimate the incidence of falls and hospitalizations due to large variance. Per protocol analyses were performed where participants in the intervention arm only included those with at least one simplification recommendation, compared to the comparison arm. Further sensitivity analyses were undertaken including only residents with two or more administration times at baseline. No a priori sample size calculation was undertaken for secondary outcomes; the original calculation for the 4 month primary outcome estimated that at least 194 residents would need to be recruited overall. All analyses were conducted using SAS Version 9.4 (SAS Institute, Cary, NC, USA).

### 2.7. Ethical Considerations

Ethical approval was provided by the Monash University Human Research Ethics Committee (0781). The study was overseen by a Project Governance Committee with weekly team meetings held to monitor study conduct. The trial was retrospectively registered with the Australian New Zealand Clinical Trials Registry in July 2017 (ACTRN12617001060336). No changes to trial methodology occurred following commencement and no interim analyses were conducted. 

## 3. Results

### 3.1. Demographics

**Figure 1:**
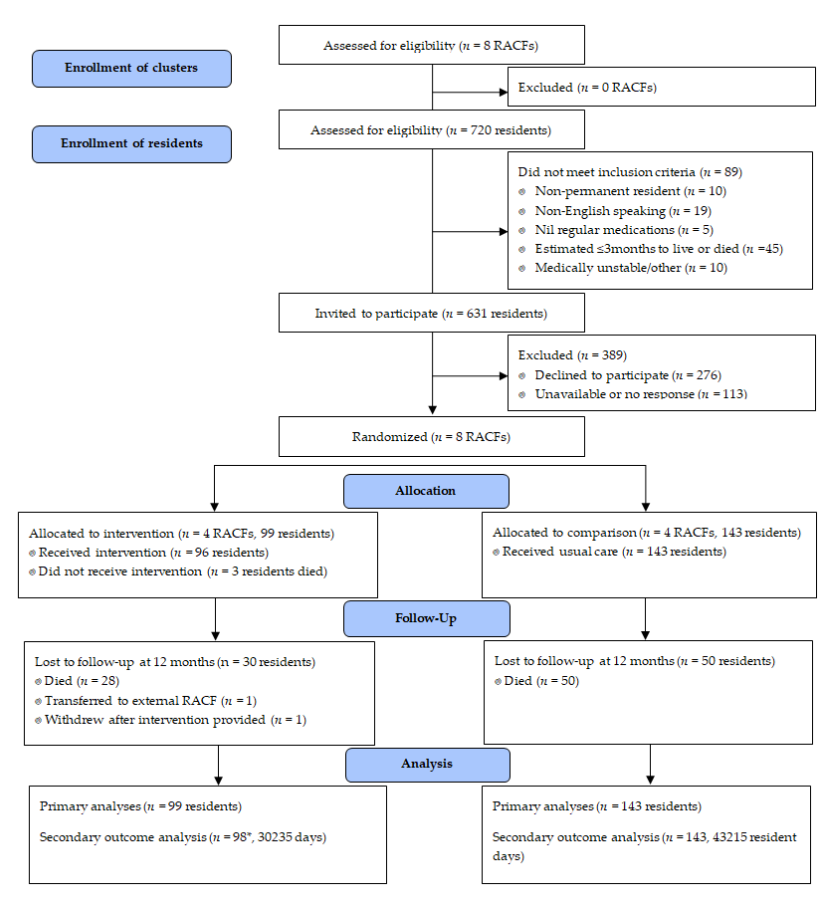
Study recruitment, enrolment and follow up to twelve months. * Falls, hospitalization and mortality data unavailable for one participant who withdrew; RACF, residential aged care facility.

Across the 8 RACFs, 242 residents were recruited including 99 from intervention facilities (Figure 1). Study participants were taking a median of nine regular daily medications (interquartile range (IQR) 6–12) (Table 1). 

Overall, the intervention was provided to 96 of the 99 residents in the intervention arm. Time to the pharmacist visit was on average 25 days (SD 13) from study entry. The pharmacist made 92 recommendations for 62 residents in the intervention arm (*n* = 65%). A comprehensive overview of the recommendations has been published elsewhere [25]. Briefly, the most common recommendation was to change the medication dose time (*n* = 60, 65.2%), followed by a change in formulation (*n* = 25, 27.2%). In almost all cases where a change to the dose time was recommended, the new administration time recommended by the pharmacist was between 0.5 and 3 h of the existing administration time. There were 133 medications implicated in the recommendations, with alimentary tract and metabolism medications (*n* = 39 recommendations), cardiovascular medications (*n* = 32), and nervous system medications (*n* = 30) most commonly implicated. Recommendations were implemented at 4 month follow up for 46 residents (57 recommendations, 62%). 

There were 162 participants (67%) remaining at 12 months, with 73,450 resident days contributed to the study (Figure 1). Baseline demographics were similar between the total cohort and remaining participants at 12 months (Table 1).

### 3.2. The Mean Number of Daily Regular Administration Times

**Figure 2:**
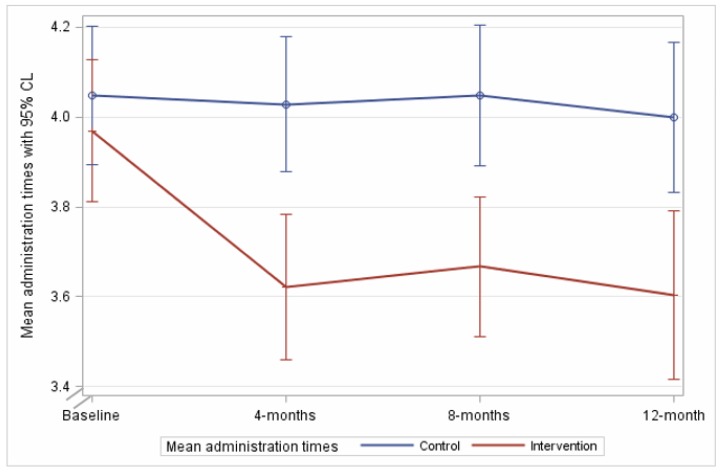
The mean number of medication administration times in 24 h during follow up in the intervention and comparison arms.

At 8 month follow up, the mean number of regular medication administration times was 3.7 (SD 1.4) in the intervention arm, and 4.1 (SD 1.7) in the comparison arm (Figure 2). A significant reduction from baseline was sustained in the intervention arm compared to the comparison arm (−0.38, 95%CI −0.69 to −0.07, *p* = 0.014). At 12 month follow up, the mean number of administration times per day for the intervention arm was 3.6 (SD 1.5), compared to 4.1 (SD 1.7) in the comparison arm, with the intervention effect sustained (−0.47, 95%CI −0.84 to −0.09, *p* = 0.014). Effect sizes were consistent in sensitivity analyses (Supplementary Table S1). The per protocol analyses including participants with at least one recommendation demonstrated a similar result at 8 months (−0.39, 95%CI −0.74 to −0.04, *p* = 0.028); this was not statistically significant at 12 months (−0.41, 95%CI −0.84 to 0.01, *p* = 0.055).

### 3.3. Falls

In the 12 months following study entry, 668 falls were reported for 140 residents (range 0–46 per resident). The intervention arm demonstrated a higher proportion of residents with at least one reported fall (71% vs. 48%, Table 2) and a higher rate of falls per 1000 resident days (13.4 vs. 5.9). This was against a background of pre-study rates of 9.4 and 9.2 per 1000 resident days in the intervention and comparison arms, respectively, for the 12 months pre-study entry.

There was a higher incidence of falls in the intervention compared to the comparison arms (IRR 2.20, 95%CI 1.33 to 3.63, *p* = 0.002). The median baseline Fall risk Assessment Tool (FRAT) scores were similar for residents in the intervention and comparison groups although differences in risk categorization were observed between study arms. Adjustment for baseline FRAT scores did not impact risk (IRR 2.24, 95%CI 1.28 to 3.93, *p* = 0.004), and effect sizes increased following sensitivity and per protocol analyses (Table 3).

Three participants had more than 20 reported falls in the 12 months post-study entry; associations remained significant after excluding these residents from analyses (IRR 2.03, 95%CI 1.26 to 3.25, *p* = 0.003), or including only resident with 10 falls or less (*n* = 226, IRR 1.66, 95%CI 1.05 to 2.66, *p* = 0.029). 

Among residents in the intervention group, stratification showed a fall rate of 15.3 per 1000 resident days in the 12 month follow up among the 36 participants where simplification was not possible, 7.7 falls per 1000 resident days among 16 residents with simplification recommendation(s) that were not implemented in the four months after study entry, and 14.0 falls per 1000 resident days in the 46 residents with at least one recommendation implemented in the four months post-study entry. 

**Figure 3:**
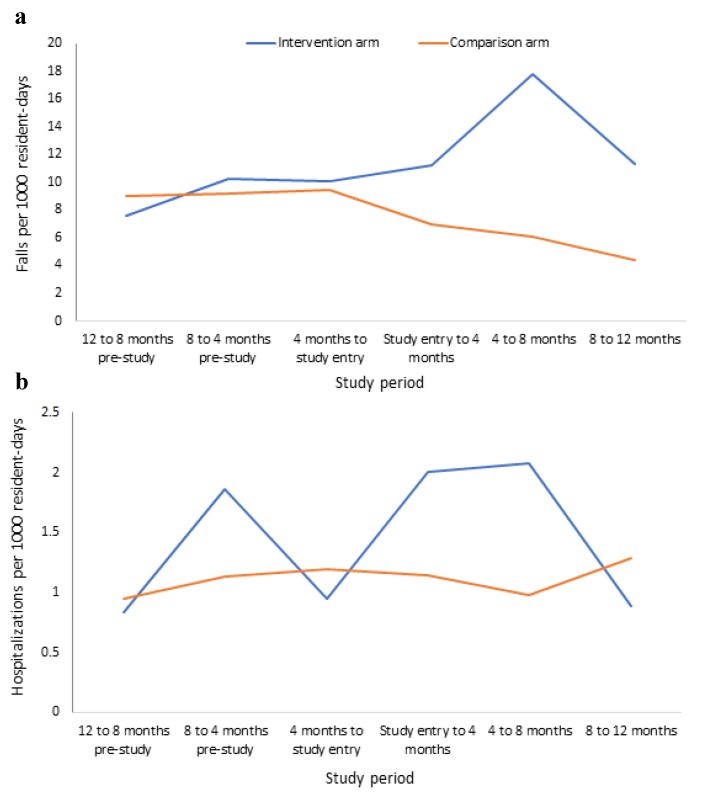
Falls (**a**) and hospitalizations (**b**) per 1000 resident days in the 12 months before and after study entry, stratified by study arm.

**Figure 4:**
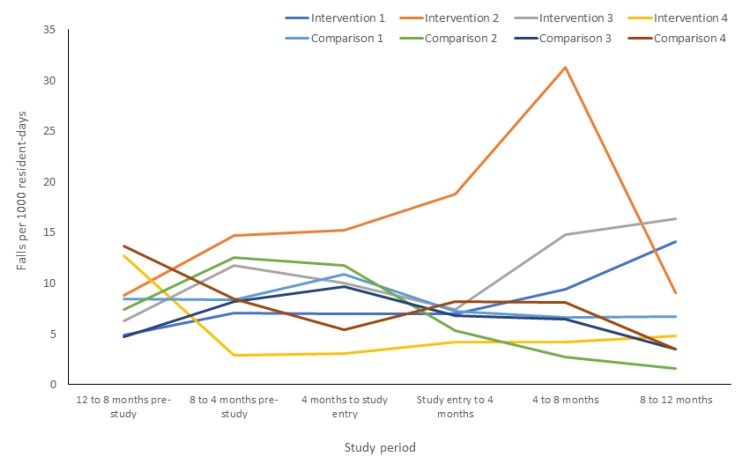
Falls per 1000 resident days in the 12 months before and after study entry, stratified by facility.

Fall rates differed between time periods, with fall rates peaking between the 4–8 month period post-study entry in the intervention arm and returning close to baseline at 12 month follow up (Figure 3). A simultaneous sustained decrease in fall rate in the intervention arm was also observed throughout follow up (Figure 3). When stratified by RACF, considerable variation in fall rates among participants in each facility was observed in the 12 months before and after study entry (Figure 4). The increase in fall rate at 4–8 month follow up in the intervention arm was primarily driven by falls at one intervention RACF, where the fall rate peaked at 31.3 falls per 1000 resident days at 4–8 month follow up and dropped to 9.1 falls per 100 resident days at 8–12 month follow up (Figure 4). This intervention RACF was matched with a comparison RACF with a very low fall rate at 12 month follow up (1.6 falls per 1000 residents).

### 3.4. Hospitalization

From study entry to 12 month follow up, the rate of hospitalizations was 1.7 per 1000 resident days in the intervention arm, compared to 1.1 in the comparison arm (Table 2). The pre-study rates were 1.2 and 1.1 hospitalizations per 1000 resident days for the intervention and comparison arms, respectively (Figure 3). The intervention was not significantly associated with hospitalization rates (IRR 1.78, 95%CI 0.57 to 5.53, *p* = 0.31; Table 3). The mean number of total days in hospital (hospitalized residents only, *n* = 65) was 7.9 (SD 13.0) in the intervention arm, compared to 7.9 days (SD 12.3) in the comparison arm, with no association with the intervention (mean difference −0.15, 95%CI −0.72 to 0.40, *p* = 0.58).

### 3.5. Mortality

Mortality was the reason for loss to follow up at 12 months for all but two participants: one withdrew, and one transferred to a facility maintained by another provider at 2 months post-study entry. Mortality rates were similar between intervention and comparison arms (28% vs. 34%, *p* = 0.32; Table 2). There were no significant differences in mortality in the intervention arm compared to the comparison arm (adjusted relative risk 0.81, 95%CI 0.81–1.38, *p* = 0.44; Table 3).

## 4. Discussion

### 4.1. Findings

In this secondary analysis of the multisite SIMPLER RCT, the reduction in the number of medication administration times observed at 4 months post-study entry was maintained at 8 and 12 months, with similar effect sizes maintained throughout follow up. This demonstrates that one-off application of a structured and validated regimen simplification tool is both effective and sustainable.

Our study addresses an important gap, as the sustainability of interventions to reduce medication regimen complexity has not been previously explored. A non-randomized trial in the United States reported significant reductions in doses and medications for hospital inpatients following provision of a visual grid depicting medication administration times to physicians [19]. In two Australian non-randomized hospital studies, ward pharmacist education led to significantly smaller increases in regimen complexity observed for participants on intervention wards at discharge compared to comparison wards [36,37]. However, participants in all three studies were predominately community dwelling (6%–20% RACF residents) and were not followed after discharge. Furthermore, evidence suggests that regular interventions reinforcing key messages are most effective for supporting sustained behavior change [38]. Future directions may include evaluation of the reproducibility of intervention effects when the intervention is provided over successive periods as it is possible that repeated simplification may further reduce administration times and other aspects of complexity.

The higher rate of falls in the intervention arm at 12 months was not anticipated, particularly as no difference in fall rates between study arms was detected at 4 month follow up [25]. A higher proportion of residents in the intervention arm experienced one or more falls in the 12 months post-study entry, despite baseline functional assessments being well matched between the study arms. The median FRAT score was similar at baseline in both study arms. However, stratification by risk categories showed that there were a higher proportion of residents classified at high risk of falls in the intervention group. The median number of regular medications that were associated with increased fall risk at baseline was similar in both study arms. However, there were differences in the composition of medications taken (i.e., lower rate of psychotropic use in the intervention arm). Fall rates during follow up varied between residents. However, post-hoc analysis showed associations remained after excluding the 15 residents with 11 or more recorded falls. Pre-study rates were similar between groups: post-intervention differences appeared driven by an increase in falls in the intervention arm, primarily at one RACF, that occurred simultaneously with a significant reduction in falls at comparison facilities. The facilities all engage in ongoing fall-minimization strategies including Fall Elimination Leadership Teams and Fall Working Groups, and no single facility-level intervention could be identified as causing the variability in rates between study arms. While risk-reporting software was uniform across all eight study institutions, incident reporting is highly dependent on staff and reporting differences between facilities may also have contributed to variation [39,40]. Furthermore, a recent aggregate root cause analysis undertaken in South Australian RACFs that examined fall-related hospitalizations over a 12 month period found that most falls that led to hospitalizations (83%) were unwitnessed [41], which could further impact on variability in fall-reporting rates. A reduction in medication administration times may lead to less points of interaction between nursing staff and residents, and less opportunity to pre-empt a resident experiencing a fall. However, due to the small effect size, a causative relationship is believed to be unlikely. The rate of falls was comparable in residents in the intervention arm who had recommendations implemented (15.3/1000 resident days) compared to residents in the intervention arm with no simplification recommendations (14.0/1000 resident days). We are not aware of any further mechanisms through which the intervention may have contributed to a higher fall rate. To our knowledge, no changes to medication administration times were considered likely to be associated with an increased fall risk for that resident. Additionally, less than half of residents in the intervention arm who experienced a fall had a simplification recommendation implemented. Facility-level activities aiming to increase resident mobility and reduce sedation (e.g., increasing allied health professional involvement) may have also inadvertently led to increases in fall risk which could be explored more in further research. Competing risk with mortality was considered unlikely to contribute, as mortality was similar between arms. We believe the higher incidence of falls in the intervention arm is likely explained by factors unrelated to and not explored in this study, including the unexplained decrease in falls in the comparison arm. We note that a 12 week exercise physiology intervention was undertaken in two of the RACFs in the comparison arm immediately prior to recruitment for the SIMPLER study and it is possible that learnings were continued to be implemented at those RACFs [42]. Further research on the association between regimen simplification and fall risk is needed. While we believe that the intervention was unlikely to have had a causal relationship with an increase in fall rate, it would be prudent to use clinical judgement and, for example, consider the possibility of falls if two medications associated with orthostatic hypotension are recommended for co-administration at the same time.

No associations were observed between the intervention and mortality or hospitalizations. The lack of studies evaluating medication regimen complexity interventions and clinical outcomes in RACFs makes comparison difficult. Previous research has reported increased mortality hazard ratios with higher regimen complexity. However, this was based on a 3 year follow up, and complexity was measured using a regimen complexity index, rather than medication administration times, limiting comparison [9]. Associations between medication complexity and hospital admission are variable in the literature, and focus predominately on community-dwelling people, though one prospective study of Australian RACF residents identified an association between increased complexity scores and hospitalization, in the absence of simplification interventions [6]. A 12 month retrospective cohort study involving 204 older Australians with chronic kidney disease who were discharged from hospital (21% were discharged to RACFs) showed that medication regimen complexity was not associated with 30 day hospital readmission, but was associated with a shorter time to readmission over 12 months [15].

### 4.2. Clinical Significance

Given the complex nature of medication regimens in residential care, small reductions in complexity at the individual resident-level that persist over 12 months have the potential to enact large changes in staff availability. Medication management is a significant component of direct care activities in RACFs and reducing time spent administering medications may enable staff to shift a meaningful period of time to other care activities or spend more time on other medication safety initiatives [40]. Previous studies have identified that time spent on medication rounds by nurses varies from approximately 1 h for 20 residents, to 4.5 h for 35 residents [43,44,45]. Data from three RACFs participating in the SIMPLER study suggests nurses spend an average of five minutes administering medications per resident per round [45]. Extrapolation of the reduction in the average number of administration times for regular medications observed at 4 month follow up suggests 85 h of staff time could be redistributed per month in a 100 bed RACF. Thus, a considerable amount of nursing time could potentially be spent on ensuring safe and high-quality medication administration practices and/or shifted to other care activities over a 12 month period.

### 4.3. Strengths and Limitations

Minor differences in baseline characteristics of residents were observed between study arms. However, adjustment in analyses did not change associations observed at 8 and 12 month follow up. The average length of stay in Australian RACFs is 30 months and the observed loss to follow up was anticipated [46]. We accounted for participant attrition by conducting constrained longitudinal data analysis for medication administration outcomes and presenting incident rates using days contributed to the study. Demographics of available participants at 12 months were predominately similar between intervention and comparison arms.

Health outcomes were assessed from study entry. However, pharmacist review occurred on average 25 days from entry and, while implementation of recommendations was collected at four month follow up, the precise date of any simplification changes was not available. Therefore, it is possible that some falls and hospitalizations occurred prior to simplification recommendations being implemented. While administration time data was collected at 8 and 12 months, further implementation of recommendations was not assessed at these time points. There is the potential that recommendations not implemented at 4 months were implemented at a later date and not captured. However, analysis was based on an intention-to-treat approach.

The eight participating sites were managed by a not-for-profit organization, which may affect replication of findings at other institutions and in the public or for-profit sectors. Similarly, while both metropolitan and regional sites were included, this trial took place in a small number of clusters and further research is required to determine applicability in other jurisdictions. The SIMPLER study intervention was delivered by a single clinical pharmacist. While MRS GRACE was designed with input from an interdisciplinary clinical panel, further research is required to demonstrate the impact of the tool when used by other health professionals.

Other potential limitations were that while participant recruitment and baseline data collection were undertaken before randomization and study allocation, residents and RACF staff were unblinded once the intervention was delivered. Finally, no a priori sample size calculation was undertaken for secondary outcomes. Because the pharmacist could have recommended simplification for any medications prescribed regularly, we examined all-cause rather than cause-specific mortality. The intervention was not anticipated to impact mortality because the pharmacist only identified opportunities to simplify the medication regimen and did not undertake a clinical review nor suggest initiation or discontinuation of any medications. Although all-cause mortality can be considered a non-specific endpoint, it is an important and commonly reported outcome in randomized controlled trials involving older people. Although investigating the association with mortality over 12 months is a relatively short follow up, this is in the context of an average length of stay in Australian RACFs of just 2.5 years [46]. For comparison, a systematic review that pooled results from 26 individualized deprescribing interventions in RACFs with a follow-up period of up to 12 months demonstrated a reduced odds of mortality post-intervention (odds ratio 0.90, 95%CI 0.82–0.99) [47]. We explored a range of secondary outcomes important to residents at the 4 month and 12 month follow ups, including falls, hospitalizations, resident satisfaction and quality of life [25]. We are also undertaking a mixed-methods process evaluation to explore the processes underpinning the implementation and uptake of the medication simplification intervention and associated experiences of stakeholders involved in the SIMPLER study [25].

### 4.4. Future Research and Implications

Medication regimen simplification using a validated, structured approach has the potential to optimize medication administration in RACFs. The sustainability of administration time reduction at 12 months suggests that an annual simplification review may be sufficient for some residents. Additional triggers for a simplification review not investigated in SIMPLER may include changes to therapeutic goals or at transitions of care such as hospital discharge, particularly as medication regimen complexity generally increases during hospitalization [18,36,37]. Results of this follow-up analysis add to the SIMPLER trial findings and support an expanded role for clinical pharmacists to work closely with physicians and nurses to optimize medication management in RACFs.

Future research is required to explore associations between medication simplification and health outcomes, including studies with adequate power to detect differences in mortality or hospitalization rates. Further exploration of potential associations between medicine regimen simplification and falls are required, due to the lack of reasonable causal relationship or existing evidence with which to contextualize our findings.

## 5. Conclusions

The impact of a one-off pharmacist simplification intervention on the number of daily administration times was sustained over 12 months. Medication simplification guided by a structured, validated tool that is designed to be applied with clinical judgement such as MRS GRACE should be considered for all residents at least annually. The findings of this study also support the role of pharmacists in interdisciplinary approaches to aged care. The higher incidence of falls in the intervention arm may be linked to unrelated factors that were not explored in this study. However, we suggest that future research could explore the impact of medication simplification on falls and advocate for the need for clinical judgement when simplifying regimens.

## Figures and Tables

**Table 1:** Baseline characteristics of participating residents at study entry and at 12 month follow up.

Characteristic at Study Entry	Intervention Baseline (*n* = 99)	Intervention 12 Months (*n* = 70)	Comparison Baseline (*n* = 143)	Comparison 12 Months (*n* = 93)
Age (years), mean (SD)	85.7 (7.8)	85.0 (7.5)	86.2 (8.3)	84.8 (8.8)
Female (*n*, %)	67 (67.7)	46 (65.7)	112 (78.3)	72 (77.4)
Regional location (*n*, %)	32 (32.3)	21 (30.0)	16 (11.2)	6 (6.5)
Length of stay in RACF (years), median (IQR)	2.3 (0.94–3.67)	2.3 (0.90–3.42)	3.7 (1.01–4.99)	2.5 (0.78–5.05)
Katz activities of daily living score, median (IQR)	1 (1–3)	2 (1–4)	1 (1–3)	2 (1–4)
FRAIL-NH score, median (IQR)	6 (3–9)	6 (2–9)	7 (3–10)	6 (2–9)
Mini Nutritional Assessment-Short Form score, median (IQR)	9 (7–11)	10 (8–11)	10 (8–11)	10 (8–11)
Charlson Comorbidity Index, median (IQR)	2 (1–4)	2 (1–3)	2 (1–3)	2 (1–3)
Fall risk Assessment Tool score				
Median overall score (IQR)	13 (11–16)	13 (11–16)	13 (11–16)	12 (10–15)
Low fall risk (*n*, %)	34 (34.3)	25 (35.7)	45 (31.5)	38 (40.8)
Medium fall risk (*n*, %)	29 (29.3)	20 (28.5)	58 (40.5)	37 (39.7)
High fall risk (*n*, %)	36 (36.3)	25 (35.7)	40 (27.9)	18 (19.3)
Diagnosed dementia (*n*, %)	54 (54.6)	38 (54.3)	77 (53.9)	44 (47.8)
Dementia severity rating scale score, median (IQR)	21 (11–39)	19 (10–36.5)	22 (12–38)	18 (10–35)
Total no. of medications charted, median (IQR)	12 (9–16)	12 (9–16)	13 (10–18)	13 (10–18)
No. of medications charted for regular use	9 (7–12)	9 (7–12)	9 (6–12)	9 (6–12)
No. of medications charted for regular daily use *	9 (6–11)	8 (6–11)	8 (5–12)	8 (5–12)
Prescribed fall risk increasing medication(s) regularly				
Median (IQR)	4 (2–6)	4 (2–6)	4 (2–6)	4 (2–6)
At least one medication (*n*, %)	95 (95.9)	68 (97.1)	138 (96.5)	90 (96.7)
At least one psychotropic (*n*, %) **	77 (77.7)	53 (75.7)	123 (86.0)	77 (82.8)
At least one medication causing orthostatic hypotension (*n*, %) ***	91 (91.9)	65 (92.8)	129 (90.2)	86 (92.4)

* Excludes nutritional drinks; SD, standard deviation; RACF, residential aged care facility; IQR, interquartile range. ** Included opioids (ATC code N02A), antipsychotics (N05A, excluding lithium (N05AN)), anxiolytics (N05B), hypnotics and sedatives (N05C) and antidepressants (N06A). *** Included vasodilators (ATC code C01D), antihypertensives (C02), diuretics (C03), beta blockers (C07), calcium-channel blockers (C08), renin angiotensin–system inhibitors (C09), alpha adrenoceptor antagonists (G04CA), dopaminergic agents (N04B), antipsychotics (N05A, excluding lithium (N05AN)) and antidepressants (N06A).

**Table 2:** Falls, hospitalizations and mortality in the intervention and control arms twelve months prior and post-study entry.

	Intervention Arm (*n* = 98 *)						Comparison Arm (*n* = 143)					
	Before Study Entry (31,790 Days)			Twelve Month Follow Up (30,235 Days)			Before Study Entry (45,436 Days)			Twelve Month Follow Up (43,215 Days)		
	No of Residents (%)	No of Events	Event Rate	No of Residents (%)	No of Events	Event Rate	No of Residents (%)	No of Events	Event Rate	No of Residents (%)	No of Events	Event Rate
Falls	57 (58.1)	300	9.4	70 (71.4)	410	13.5	88 (61.5)	421	9.2	70 (48.9)	258	5.9
Hospitalizations	26 (26.5)	39	1.2	29 (29.5)	52	1.7	38 (26.5)	50	1.1	36 (25.1)	49	1.1
Mortality	-	-	-	28 (28.5)	-	-	-	-	-	50 (34.5)	-	-

* Follow-up data unavailable for one participant who withdrew; event rate per 1000 resident days.

**Table 3:** Adjusted differences in health outcomes in the intervention versus comparison arms at 12 months post-study entry.

Model	Falls		Hospitalizations		Mortality	
	IRR (95%CI), Intervention vs. Control	*p*-Value	IRR (95%CI), Intervention vs. Control	*p*-Value	RR (95%CI), Intervention vs. Control	*p*-Value
All participants, RACF as random effect (*n* = 241 *)	2.20 (1.33–3.63)	0.002	1.78 (0.57–5.53)	0.31	0.81 (0.48–1.38)	0.44
All participants, with pre-rate #, plus RACF as random effect (*n* = 241)	2.43 (1.50–3.93)	<0.001	1.78 (0.71–4.52)	0.21	--	--
All participants, age, gender, regional, LOS, CCI; random RACF, (no pre-rate) (*n* = 241)	2.43 (1.61–3.63)	<0.001	2.03 (0.75–5.53)	0.16	0.76 (0.50–1.15)~	0.20
All participants, age, gender, regional, LOS, CCI; with pre-rate and random RACF (*n* = 241)	2.61 (1.73–3.93)	<0.001	1.84 (0.78–4.39)	0.16	--	--
Residents with ≥ 2 admin times, with pre-rate, random RACF (*n* = 235)	2.48 (1.52–4.05)	<0.001	1.71 (0.63–4.75)	0.29	0.83 (0.50 to 1.38)^	0.47
Intervention residents with ≥ 1 recommendation, with pre-rate, random facility (*n* = 205)	2.55 (1.49–4.34)	<0.001	1.71 (0.60–4.90)	0.31	0.79 (0.41 to 1.50)^	0.47

* Follow-up data unavailable for one participant who withdrew; # pre-rate of relevant incidents in the twelve months prior to study entry date; IRR, incident rate ratio; CI, confidence intervals; RR, relative risk; RACF, residential aged care facility; LOS, length of stay; CCI, Charlson Comorbidity Index; ~, without random facility; ^, no pre-rate.

## Supplementary Materials

The following are available online at https://www.mdpi.com/2077-0383/9/4/1053/s1, Table S1: Change in the number of medication administration times from study entry to 12 month follow up, in the intervention arm compared to the comparison arm.
