# Supplementary material for: Impact of Medication Regimen Simplification on Medication Administration Times and Health Outcomes in Residential Aged Care: 12 Month Follow Up of the SIMPLER Randomized Controlled Trial
Source: J Clin Med. 2020 Apr 8;9(4):1053. doi: 10.3390/jcm9041053 (PMC7231224; doi:10.3390/jcm9041053)
Supplement: Supplementary file 1 [file jcm-09-01053-s001.pdf]

Supplementary Table S1: Change in number of medication administration times from study entry to 12-month follow-up, in intervention arm compared to comparison arm.

	Change, mean difference (95% confidence intervals), compared to baseline		
	4-month follow-up	8-month follow-up	12-month follow-up
**Constrained longitudinal data analysis (cLDA) model**			
All participants, with RACF as a random effect	-0.36 (-0.63, -0.09) $p = 0.010$	-0.38 (-0.69, -0.07) $p = 0.014$	-0.47 (-0.84, -0.09) $p = 0.014$
All participants, with RACF, age, sex, CCI and LOS as fixed effects	-0.35 (-0.62, -0.08) $p = 0.011$	-0.37 (-0.68, -0.06) $p = 0.018$	-0.46 (-0.83, -0.08) $p = 0.016$
Residents with ≥ 2 administration times at baseline ($n = 235$)	-0.37 (-0.64, -0.09) $p = 0.009$	-0.36 (-0.66, -0.05) $p = 0.022$	-0.43 (-0.81, -0.05) $p = 0.024$
Intervention arm included residents with ≥ 1 simplification recommendation ($n = 205$)	-0.49 (-0.80, -0.18) $p = 0.001$	-0.39 (-0.74, -0.04) $p = 0.028$	-0.41 (-0.84, 0.01) $p = 0.055$

RACF: residential aged care facility; CCI: Charlson Comorbidity Index; LOS: length of stay
